# Gene Profile of Chemokines on Hepatic Stellate Cells of Schistosome-Infected Mice and Antifibrotic Roles of CXCL9/10 on Liver Non-Parenchymal Cells

**DOI:** 10.1371/journal.pone.0042490

**Published:** 2012-08-08

**Authors:** Yue-jin Liang, Jie Luo, Qiao Lu, Ying Zhou, Hai-wei Wu, Dan Zheng, Yong-ya Ren, Ke-yi Sun, Yong Wang, Zhao-song Zhang

**Affiliations:** 1 Department of Pathogen Biology, Key Laboratory of Pathogen Biology of Jiangsu Province, Nanjing Medical University, Nanjing, Jiangsu, China; 2 Center for International Health Research, Rhode Island Hospital, Brown University, Providence, Rhode Island, United States of America; 3 Department of Pathology, Nanjing Medical University, Nanjing, Jiangsu, China; 4 Department of Microbiology & Immunology, Nanjing Medical University, Nanjing, Jiangsu, China; Queensland Institute of Medical Research, Australia

## Abstract

Hepatic stellate cells (HSCs) play a key role in the development of liver fibrosis caused by schistosomiasis. Chemokines were widely expressed and involved in cellular activation, proliferation and migration in inflammatory and infectious diseases. However, little is known about the expressions of chemokines on HSCs in the schistosoma infection. In addition, the roles of chemokines in pathogenesis of liver fibrosis are not totally clear. In our study, we used microarray to analyze the temporal gene expressions of primary HSCs isolated from mice with both acute and chronic schistosomiasis. Our microarray data showed that most of the chemokines expressed on HSCs were upregulated at 3 weeks post-infection (*p.i*) when the egg granulomatous response was not obviously evoked in the liver. However, some of them like CXCL9, CXCL10 and CXCL11 were subsequently decreased at 6 weeks *p.i* when the granulomatous response reached the peak. In the chronic stage, most of the differentially expressed chemokines maintained persistent high-abundances. Furthermore, several chemokines including CCR2, CCR5, CCR7, CXCR3, CXCR4, CCL2, CCL5, CCL21, CXCL9 and CXCL10 were expressed by HCSs and the abundances of them were changed following the praziquantel treatment in the chronic stage, indicating that chemokines were possibly necessary for the persistence of the chronic stage. In vitro experiments, hepatic non-parenchymal cells, primary HSCs and human HSCs line LX-2 were stimulated by chemokines. The results showed that CXCL9 and CXCL10, but not CXCL11 or CXCL4, significantly inhibited the gene expressions of Col1α1, Col3α1 and α-SMA, indicating the potential anti-fibrosis effect of CXCL9 and CXCL10 in schistosomiasis. More interestingly, soluble egg antigen (SEA) of *Schistosoma japonicum* was able to inhibit transcriptional expressions of some chemokines by LX-2 cells, suggesting that SEA was capable of regulating the expression pattern of chemokine family and modulating the hepatic immune microenvironment in schistosomiasis.

## Introduction

The immunopathological damage in schistosomiasis japonica is mainly due to the granulomatous inflammation around parasite eggs in host liver during acute phase, which may result in liver fibrosis in the chronic phase and finally lead to death [1], [2]. Hepatic stellate cells (HSCs) play a key role in process of liver fibrosis caused by different diseases [3]. Following liver injury, HSCs undergo a response known as “activation”, the transition from quiescent cells into proliferative, fibrogenic and contractile myofibroblasts [4]. Activated HSCs secrete excessive extracellular matrix (ECM) which is deposited among the hepatocytes, resulting in liver fibrosis [3]. Several cytokines and chemokines are produced by activated HSCs and involved in progressive liver fibrosis [3], [5].

Recently, researches reveal that chemokines and their receptors are involved not only in cellular migration but also in activation and proliferation of HSCs in both acute and chronic liver diseases [6], [7]. HSCs express several chemokines and receptors as described in animal model of diseases and clinical observations [8], [9], [10], [11]. Some chemokine receptors of HSCs, such as CCR1, CCR2 and CCR5, are considered to be pro-fibrosis targets [8], while CXCR3 associated chemokine CXCL9 is described to be an anti-fibrosis cytokine [10]. However, the roles of chemokines and receptors on HSCs in mice of schistosomiasis are not totally clear. Therefore, the features and functions of the entire chemokine family on HSCs in different stages of liver disease are worth investigating. In addition, schistosoma egg-induced downregulation of HSCs activation and fibrogenesis has been reported [12], but whether egg antigens would affect the expression of chemokines on HSCs is still unknown.

In this study, we isolated primary HSCs from mice infected with *Schistosoma japonicum* (*S. japonicum*) in normal phase (0 weeks), phase before eggs laid (3 weeks), acute phase with severe granulomatous response (6 weeks), chronic phase (12 weeks) and advanced phases (18 weeks). Then we used microarray to analyze the dynamic gene expressions of HSCs and described the feature of chemokines on HSCs in different stages of schistosomiasis. We treated mice infected with *S. japonicum* with praziquantel (PZQ), which is an anti-parasite drug with anti-fibrosis effect [13], to study the roles of chemokines on HSCs after the successful pathogenic cure. After bio-function analysis, we focused on two chemokines CXCL9 and CXCL10, and showed that both of them can inhibit the expression of collagen in human HSCs line LX-2, liver non-parenchymal cells, and primary HSCs of schistosomiasis mice indicating the anti-fibrosis property. In addition, LX-2 was also stimulated with *S. japonicum* soluble egg antigen (SEA) to reveal the effect of parasite-origin products on gene expressions of some chemokines and receptors on HSCs.

## Materials and Methods

### Animal Model

A total of 70 female BABL/c mice weighing 15 to 25 g were purchased from the Comparative Medicine Center of Yangzhou University (Jiangsu, China) and maintained in the Animal Center of Nanjing Military Medicine Institute (Jiangsu, China) according to guidelines approved by the Nanjing Medical University Animal Experiment and Care Committee. Mice were infected by invasion of peritonaeum with 12±2 cercarias of *S. japonicum*, which were obtained from infected snails, provided by Jiangsu Institute of Parasitic Diseases (Jiangsu, China). Mice of 3, 6, 12 and 18 weeks post-infection (*p.i*) were sacrificed and normal mice were adopted as control. In addition, PZQ (Sigma-Aldrich, St. Louis, MO), was resuspended in 1% carboxymethylcellulose solution and administrated to another two groups of infected mice at 6 and 12 weeks post-infection by gavage for 3 days (250 mg/kg body weight/day). These two groups of PZQ-treated mice were kept for another 6 weeks (12 weeks and 18 weeks post-infection respectively) until sacrificed. Each group contained ten mice.

### H&E and Sirius Red Staining

Mice livers were fixed in 10% neutralized formaldehyde and embedded in paraffin. Tissue sections (4-µm thick) were stained with H&E (hematoxylin and eosin) and aqueous saturated solution of picric acid containing 0.1% Sirius Red (Sigma-Aldrich, St. Louis, MO). Images of six random microscopic fields in the liver section of each mouse were recorded by using an inverted microscope (ZEISS, Goettingen, Germany), and then digitized and analyzed in Image-Pro Plus software as previously described [14]. Single egg granulomas were highlighted in black cycles in the histochemical pictures.

### Isolation of Mice Primary HSCs and Non-parenchymal Cells

HSCs were isolated from mice by the modified method as described [15]. Briefly, in situ perfusion of the liver was initiated with DMEM (Hyclone, Thermo Fisher Scientific, Beijing, China), followed by perfusion with DMEM containing 0.04% collagenase type IV and 0.2% pronase (Gibco Life Technologies, Grand Island, NY, USA) at 37°C for 10 minutes. Then, the liver was further digested with DMEM containing 0.08% collagenase type IV, 0.08% pronase and 10U/ml DNase I (Sigma-Aldrich, St. Louis, MO) at 37°C bath shaking for 30 minutes. 10% and 28% Optiprep (Axis-Shield PoC AS, Oslo, Norway) were used respectively for density gradient centrifugations of HSCs and non-parenchymal cells [16], [17]. Purity of HSCs was estimated based on the autofluorescence. Cell viability was examined by Trypan blue exclusion [18]. Both cell purity and viability were in excess of 90%. HSCs, isolated from each group of ten mice were mixed as one sample for the microarray experiments. Hepatic non-parenchymal cells, which were much smaller than hepatic parenchymal cells, were detected under light microscope and the purity was higher than 99%.

### Gene Expression and Analysis on Affymetrix GeneChip® Mouse Genome 430A 2.0 Arrays

Total RNA was extracted immediately from freshly isolated HSCs of different groups by using Trizol reagent (Invitrogen, Carlsbad, CA) according to manufacturer’s instructions, and digested with DNase I at 37°C for 15 min to remove any contaminating DNA. The RNA was cleaned up with RNeasy Kit (Qiagen, Hilden, Germany) and the quantities and qualities were determined by spectrophometer and 1% formaldehyde denaturing gel electrophoresis. The samples with bright bands of ribosomal 28S to18S RNA in a ratio >1.5∶1 were used for microarray analysis. Affymetrix GeneChip® Mouse Genome 430A 2.0 array (Affymetrix, Santa Clara, CA), which includes approximately 14,000 annotated genes with over 22,600 probe sets from the mouse genome, was used in microarray analysis. Hybridization, data capture, and analysis were performed by CapitalBio Corporation (a service provider authorized by Affymetrix Inc, Beijing, China). Briefly, 100 ng of total RNA was used for cDNA synthesis in order to produce biotin-tagged cRNA with GeneChip IVT Labeling kit (Affymetrix, Santa Clara, CA). 10 µg fragmented cRNA, with control oligo B2 and eukaryotic hybridization controls (bioB, bioC, bioD, cre) was hybridized to each GeneChip array at 45°C for 16 hours (Affymetrix GeneChipHybridization Oven 640) according to manufacturer’s instructions. After hybridization, the GeneChip arrays were washed, and then stained with streptavidin phycoerythrinonan (SAPE) with Affymetrix Fluidics Station 450 followed by scanning with the Affymetrix GeneChip Scanner 3000 7G. The data were analyzed in GeneChip Operating software (GCOS 1.4). A cut-off of 2-fold changes in expression was used allowing identification of changes in the gene expression. The genes of differential expression were clustered by using Cluster 3.0 software. However, the signal-absent genes were not used in the cluster because they would confuse the functional analyses. Therefore, the individual gene, which was signal-absent in more than 2 groups was considered as expression-absent gene and filtered out in the cluster analyses. Hierarchical clustering was performed using Pearson correlation. The name of the differential genes from the cluster were input into Ingenuity Pathway Analysis (Ingenuity Systems, INC., Redwood City, CA) for the analyses of biological functions and metabolic or signaling pathways. Data of microarray have been uploaded in ArrayExpress (accession number: E-MEXP-3441).

### Gene Expression of Chemokines and Receptors on Primary HSCs by QuantiGene Plex 2.0 Reagent System

Target-specific RNA molecules (CCL2, NM_011333; CCL3, NM_011337; CCL4, NM_013652; CCL7, NM_013654; CXCL9, NM_008599; CXCL10, NM_021274; CCL21, NM_011335; CCR1, NM_009912; CCR2, NM_009915; CCR5, NM_009917; CCR7, NM_007719; CXCR4, NM_009911) of primary HSCs were detected by QuantiGene Plex 2.0 Reagent System as manufacturer’s protocol (Affymetrix, Fremont, CA). Briefly, RNA from cell lysis was captured by fluorescent microspheres. Signals of cascade amplification were detected by Luminex 100 xMAP technology and Bio-Plex 5.0 software (Bio-Rad Laboratories, Hercules, CA). The geometric means of three housekeeping genes (GAPDH, NM_008084; PPIB, NM_011149; HPRT1, NM_013556) were used in each sample for normalizations. Fold-Changes were the relative ratios between normalized values of four infected groups and that of the normal group. The HSCs from 3–5 mice in each group were mixed together for one sample to reduce the individual difference. Correlation between quantigene and microarray data was caculated in GraphPad Prism Version 4.0 (GraphPad Software, San Diego, USA) using Spearman’s Rho measure.

### Preparation of *S. japonicum* Soluble Egg Antigen (SEA) and Stimulations of Cells by Chemokines and SEA *in vitro*

Freeze-dried *S. japonicum* eggs were homogenized in phosphate-buffered saline (1∶50w/v), then the homogenates were placed at 4°C for 3 days with occasional shake. The prepared homogenates were centrifuged at 4°C, 10000 g, 25 mins and supernatants were isolated as SEA.

LX-2 was obtained from Central South University (China) and cultured as described [19]. LX-2 was starved for 12 hours with DMEM of 0.2% FCS for subsequent experiments. SEA of different concentrations (0, 5, 10, 20 µg/ml, which approximately correspond with 0, 500, 1000, 2000 eggs [12]) were co-cultured with LX-2 for 24 hours. LX-2 was also activated by TGF-β(2.5 ng/ml)for 12 hours, followed by co-culture with human recombinant CXCL9, CXCL10, CXCL11 and CXCL4 (2 or 4 µg/ml respectively) for another 24 hours as other report [10]. Hepatic non-parenchymal cells which were mainly composed of infiltrative lymphocytes, macrophages and eosinophils, were all isolated from mice infected with *S.japonicum* for 12 weeks. HSCs were also isolated from the mice of 12 weeks *p.i*. Freshly isolated cells were cultured using DMEM with 10%FBS overnight and then stimulated with mouse recombinant CXCL9 or CXCL10 of various concentrations (0.1, 0.2, 0.5 or 1 µg/ml) for 24 hours [10]. Cytokines were purchased from Peprotech, USA.

### RNA Extraction and Real Time PCR

Cells were lysed in Trizol and RNA was extracted according to the manufacturer’s protocol. The quantities and qualities of RNA were determined by spectrophometer and 1% formaldehyde denaturing gel electrophoresis. Reverse transcriptase (RT) reactions were carried out by the use of RevertAid™ First Strand cDNA Synthesis Kit with oligo-dT primer (Fermentas, EU). Relative expression of RNA was determined by Real Time PCR with SYBR Green PCR Mix (Roche Diagnostics, Indianapolis, IN, USA) by using ABI7300. The primers (Table S1) were obtained from PrimerBank (http://pga.mgh.harvard.edu/primerbank/) and synthesized by Invitrogen. Cycling conditions were 2 min at 50°C, then 10 min at 95°C and followed by 40 circles of 95°C for 15 s and 60°C for 1 min. Data were normalized to housekeeping gene GAPDH and results were expressed as fold amplifications [20]. Each experiment was repeated three times.

### Statistical Analysis

T-test (two tails) and One-Way ANOVA (Newman-Keuls Multiple Comparison Test for analysis of two groups) were used in our statistical analysis. These analyses were performed by the use of the GraphPad Prism Version 4.0.

## Results

### Evaluation of Liver Egg Granuloma and Fibrosis

**Figure 1:**
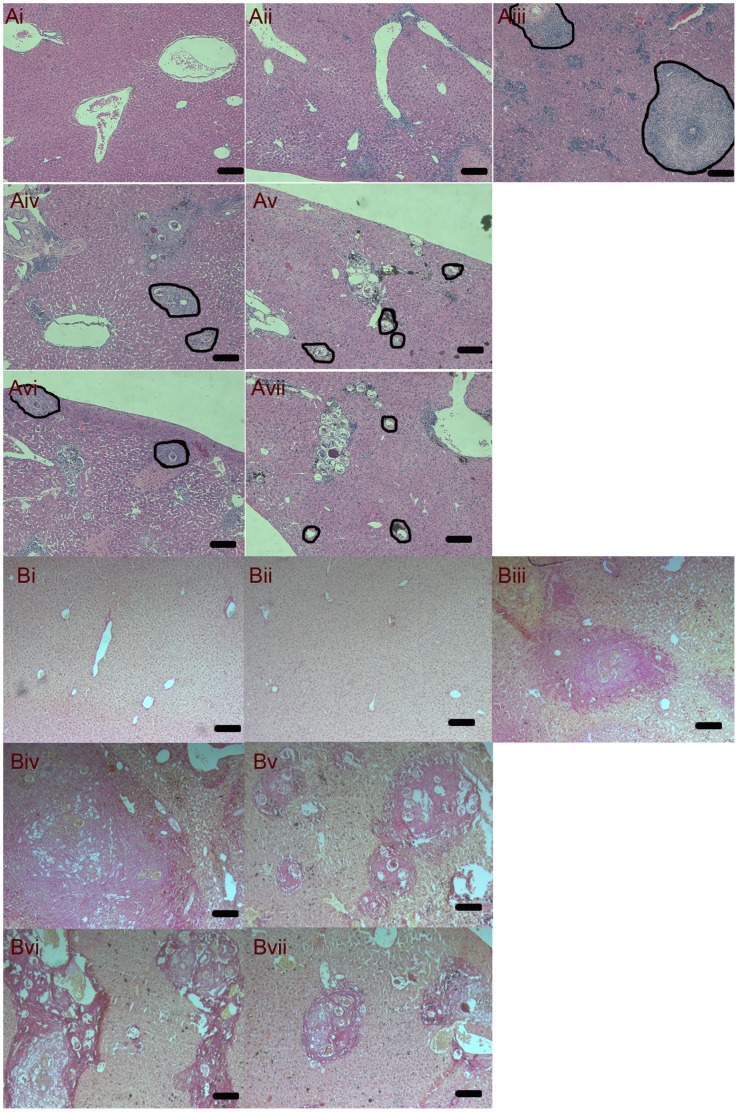
Representative images of hepatic H&E staining for normal (Ai), 3 weeks *p.i* (Aii), 6 weeks *p.i* (Aiii), 12 weeks *p.i* (Aiv), 18 weeks *p.i* (Av), 12 weeks PZQ (Avi) and 18 weeks PZQ (Avii) mice. Single egg granulomas were cycled for areas measurement by Image-Pro Plus software and highlighted in black cycles. Bar is 100 µm. Representative images by light microscope (×100) of hepatic Sirius Red staining for normal (Bi), 3 weeks *p.i* (Bii), 6 weeks *p.i* (Biii), 12 weeks *p.i* (Biv), 18 weeks *p.i* (Bv), 12 weeks PZQ (Bvi) and 18 weeks PZQ (Vii) mice.

In order to evaluate the hepatic pathology in different stages of mice infected with *S. japonicum*, H&E staining and Sirius Red staining of liver sections were performed. In 3 weeks *p.i*, there was no visible egg in the liver, but the mild infiltration of inflammatory cells was observed (Figure 1.Aii). The typical egg granulomas were visible in the acute stage of 6 weeks *p.i* (Figure 1.Aiii). In the chronic (12 weeks *p.i*, Figure 1. Aiv and Biv) and advanced (18 weeks *p.i*, Figure 1. Av and Bv) stages, plenty of collagen were accumulated in the liver, while the size of granulomas was gradually reduced. These temporal changes of hepatic pathology were consistent with previous description in mouse schistosomiasis [1]. Pathological sections also showed the decreased size of liver egg granulomas in 12 weeks PZQ group (Figure 1. Avi) compared with those of 12 weeks *p.i* mice (Figure 1.Aiv, P<0.05) and the reduced areas of collagen in 12 and 18 weeks PZQ group (Figure 1. Bvi and Bvii) compared with those of 12 and 18 weeks *p.i* mice (Figure 1. Biv and Bv, P<0.05). The data analyses were showed in Table S2. These results indicated that administration of PZQ can reduce the severity of immunopathogenesis, especially in chronic stages of schistosomiasis.

### Microarray Analysis of HSCs from Schistosomiasis Mice

**Figure 2:**
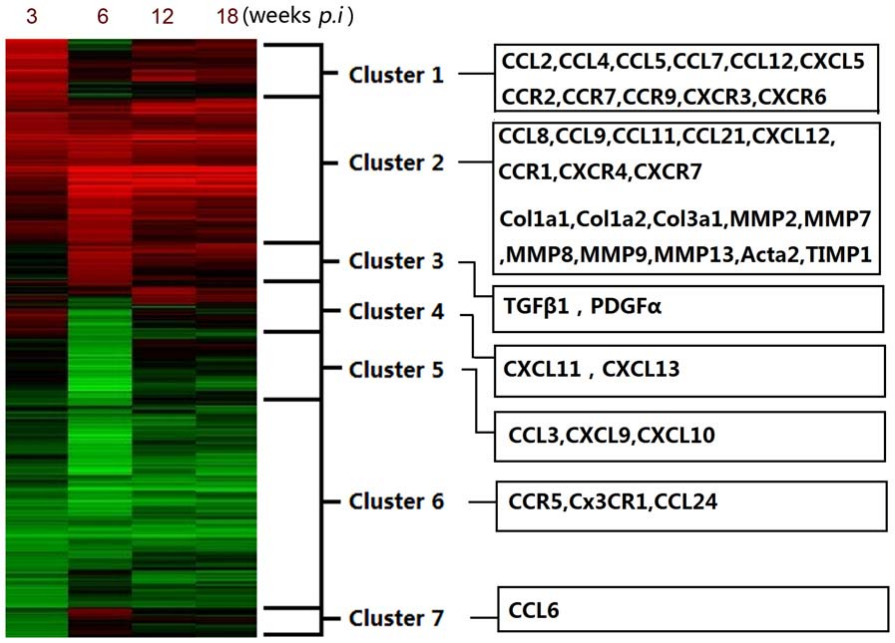
Temporal gene expressions of primary HSCs in schistosomiasis mice were divided to seven different clusters with distinct biological functions. Gene expression is represented as a heat map with relatively unchanged genes coloured black, down regulated genes coloured green and up-regulated genes coloured red. Upper numbers of the map denote the 3, 6, 12, 18 weeks *p.i* groups. Chemokines and fibrosis associated genes were listed in each box.

In this experiment, we would like to use the microarray of high-throughput technology to provide an integral view of chemokines family expressed on HSCs in several stages of infection. After the normalization of data, 6505 genes were differentially expressed. According to Hierarchical clustering, differentially expressed genes were divided into seven clusters (Figure 2). Biological functions and signaling pathways for each cluster were exhibited (Table S3 and Table S4). The analyses showed that the biological functions about inflammatory response, cancer, immunological disease, organismal injury/abnormalities and infectious disease were on the top of the list. Meanwhile, the signal pathways about cell cycle, oxidative phosphorylation, metabolism, acute phase response and antigen presentation were significantly changed during the infection. Key word “chemokine” was used to find out the chemokine associated genes and their temporal expressions were listed (Table 1). As shown in Figure 2, several chemokines and receptors such as CCL2, CCL4, CCL5, CCL7, CCL12, CCR2, CCR7, CCR9 and CXCR3 were located in cluster 1, which were up-regulated at 3 weeks *p.i*. Compared with 3 weeks *p.i,* these genes were down-regulated at 6 weeks *p.i*, and subsequently kept high level during chronic stages. There were also several persistent high-expressed chemokines and receptors such as CCL8, CCL9, CCL11, CCL21, CXCL12, CCR1, CXCR4 and CXCR7 in cluster 2, which showed a similar pattern of expression with fibrosis associated genes Col1, Col3, ATCA2 and MMP/TIMP. Some others such as CCL3, CCR5, CXCL9, CXCL10, CX3CR1 and CCL24 showed a low expression during the infection.

**Table 1:** Expression of chemokines and receptors by hepatic stellate cells in different stages of schistosomiasis.

Chemokine Symbol	Probe ID	Accession #	3 weeks	6 weeks	12 weeks	18 weeks
Ccl1	1421688_a_at	NM_011329	3.8	–	–	–
Ccl2	1420380_at	AF065933	2.6	0.6	2.2	1.5
Ccl3	1419561_at	NM_011337	1.8	0.4	2.2	1.6
Ccl4	1421578_at	AF128218	2.4	0.7	1.7	1.2
Ccl5	1418126_at	NM_013653	33.3	1.4	15.3	7.8
Ccl6	1417266_at	BC002073	0.8	1.0	1.0	1.1
Ccl7	1421228_at	AF128193	4.0	1.1	2.6	2.0
Ccl8	1419684_at	NM_021443	8.1	37.1	15.1	18.1
Ccl9	1448898_at	AF128196	2.5	3.2	11.9	11.3
Ccl11	1417789_at	NM_011330	2.8	8.0	3.5	3.7
Ccl12	1419282_at	U50712	3.0	1.0	2.1	1.0
Ccl17	1419413_at	NM_011332	8.0	–	–	–
Ccl19	1449277_at	NM_011888	–	–	–	–
Ccl20	1422029_at	AF099052	–	–	–	–
CcL21	1419426_s_at	NM_011335	3.2	11.8	11.5	22.2
Ccl22	1417925_at	BC012658	9.9	–	–	2.5
Ccl24	1450488_at	AF281075	0.2	0.3	0.2	0.3
Ccl25	1418777_at	NM_009138	–	–	–	–
Ccl27	1419188_s_at	NM_011336	–	–	–	–
Ccl28	1450217_at	BG867337	–	–	–	–
Cxcl1	1419209_at	NM_008176	0.9	0.9	1.0	1.0
Cxcl2	1449984_at	NM_009140	1.1	0.9	1.1	1.1
Cxcl5	1419728_at	NM_009141	76.6	4.4	11.4	21.6
Cxcl9	1418652_at	NM_008599	2.1	0.0	0.9	0.4
Cxcl10	1418930_at	NM_021274	2.0	0.1	1.6	0.6
Cxcl11	1419697_at	NM_019494	5.1	0.1	1.7	0.5
Cxcl12	1448823_at	BC006640	1.3	2.6	2.0	2.5
Cxcl13	1448859_at	AF030636	1.0	0.3	3.5	1.3
Cxcl14	1418456_a_at	AF252873	1.3	1.6	1.2	1.2
Cxcl15	1421404_at	NM_011339	–	–	–	–
Cxcl16	1418718_at	BC019961	0.6	1.1	1.2	1.2
Cxcl17	1451610_at	BC024561	–	–	–	–
Xcl1	1419412_at	NM_008510	1.8	1.1	1.2	1.2
Cx3cl1	1415803_at	AF010586	–	–	–	–
Ccr1	1419609_at	AV231648	8.9	12.6	20.7	17.7
Ccr2	1421186_at	BB148128	8.3	0.4	2.2	1.4
Ccr3	1422957_at	NM_009914	0.1	–	–	–
Ccr4	1421655_a_at	NM_009916	–	–	–	–
Ccr5	1422259_a_at	X94151	1.0	0.1	0.4	0.2
Ccr6	1450357_a_at	NM_009835	3.9	–	0.5	1.7
Ccr7	1423466_at	BB204380	10.3	–	1.6	4.8
Ccr8	1422291_at	NM_007720	–	–	–	–
Ccr9	1427419_x_at	AJ131357	13.2	–	0.3	3.4
Ccr10	1421420_at	AF215982	–	–	–	–
Cxcr2	1421734_at	NM_009909	26.4	–	15.1	–
Cxcr3	1449925_at	NM_009910	4.2	0.4	0.6	0.8
Cxcr4	1448710_at	D87747	1.7	1.3	2.3	2.9
Cxcr5	1422003_at	NM_007551	2.8	–	1.1	2.6
Cxcr6	1422812_at	NM_030712	7.9	0.1	1.4	1.5
Cxcr7	1417625_s_at	BC015254	3.7	1.4	5.7	5.4
Cx3cr1	1450020_at	BC012653	0.3	0.2	0.2	0.2

Expression values are generated from microarray data and are displayed as a ratio relative to uninfected mice.

-equals not detected.

### Expression Changes of Chemokines and Receptors on HSCs After PZQ Treatment

It is demonstrated that administration of PZQ could improve the damage of immunopathogenesis, while the changes of expression of chemokines family on HSCs after PZQ treatment would be interesting. The diversity may reveal the regulatory mechanisms of HSCs by chemokines and their receptors. As shown in Table 2, compared with PZQ un-treated groups of the same time-point infected mice, several chemokines were up-regulated after PZQ treatment, including CCL3, CCL4, CCL5, CCL17, CCL22, CCL24, CXCL9, CXCL10 and CXCL11. Meanwhile, most of the receptors including CCR2, CCR3, CCR5, CCR6, CCR7, CXCR3, CXCR5, CXCR6 and XCR1 were also up-regulated. However, CCL8, CCL9, CCL11, CCL21, CXCL5, CXCL12 and CXCL13 were down-regulated in PZQ treatment groups as well as receptors CCR1, CXCR4 and CXCR7. CCL2, CCL7 and other night members CCL1, CCL6, CCL12, CXCL1, CXCL2, CXCL14, CXCL16, XCL1 and CX3CR1 showed no change after PZQ treatment.

**Table 2:** Gene array expression of chemokines and receptors by hepatic stellate cells in PZQ-treated mice of schistosomiasis.

Symbol	12weeks PZQ	18weeks PZQ	Symbol	12weeks PZQ	18weeks PZQ	Symbol	12weeks PZQ	18weeks PZQ
*Up-regulated*			*Down-regulated*			*Not changed*		
Ccl3	2.2	2.3	Ccl8	0.2	0.3	Ccl1	1.8	1.5
Ccl4	2.3	1.9	Ccl9	0.3	0.1	Ccl2	1.4	0.6
Ccl5	1.9	3.5	Ccl11	–	–	Ccl6	1.1	0.8
Ccl17	1.6	2.0	CcL21	0.2	0.1	Ccl7	1.6	0.5
Ccl22	5.7	0.9	Cxcl5	0.9	0.1	Ccl12	1.5	1.0
Ccl24	8.2	3.3	Cxcl12	0.3	0.4	Cxcl1	1.4	1.0
Cxcl9	1.9	6.0	Cxcl13	0.4	1.4	Cxcl2	1.2	1.0
Cxcl10	3.4	3.6	Ccr1	0.1	0.6	Cxcl14	1.0	0.6
Cxcl11	2.2	7.1	Cxcr4	0.4	1.2	Cxcl16	0.8	0.6
Ccr2	0.9	3.7	Cxcr7	0.1	0.2	Xcl1	1.0	1.0
Ccr3	16.3	10.9				Cx3cr1	1.6	1.1
Ccr5	1.2	2.3						
Ccr6	2.8	3.1	*Not consistent*					
Ccr7	2.5	2.1	Cxcr2	–	10.4			
Ccr9	16.2	3.8						
Cxcr3	4.3	5.9						
Cxcr5	3.0	16.9						
Cxcr6	2.4	2.4						
Xcr1	2.1	2.5						

Expression values are generated from microarray data.

The ratio of 12, 18 weeks PZQ-treated mice are relative to concurrent infected mice.

-equals not detected.

### Validation of Chemokines and Receptors Expression on HSCs

**Figure 3:**
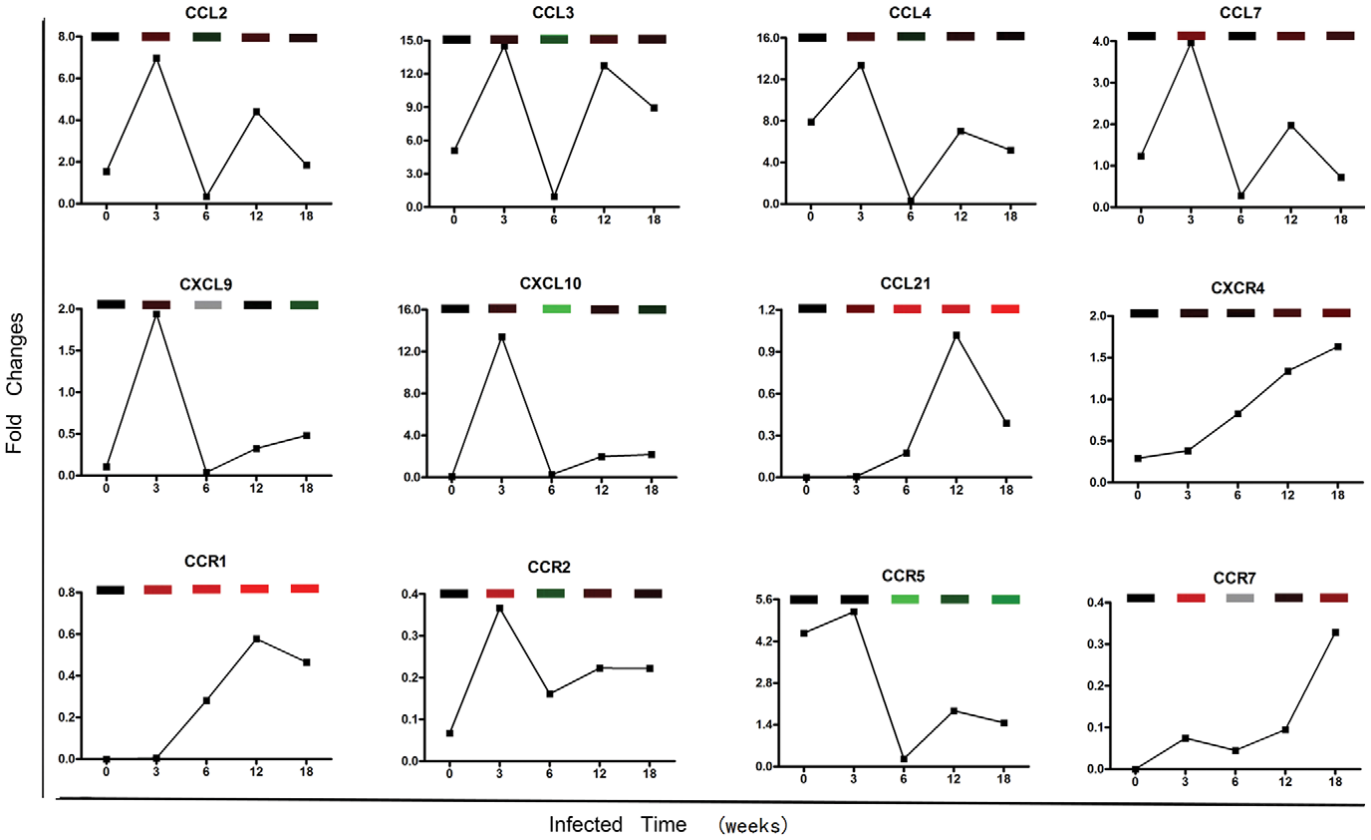
Part of genes associated with chemokines in microarray data were validated by QuantiGene Plex 2.0 Reagent System. The curves were showed the gene expression in 0, 3, 6, 12, 18 weeks *p.i* groups. The above bricks were the temporal gene expressions in microarray. Gene expression is represented as heat map with relatively unchanged genes coloured black, down regulated genes coloured green, up-regulated genes coloured red and no signal genes coloured grey. Each sample of individual time-point is from more than 6 mice. The assay is normalized by three housekeeping genes and operated by duplicates. The relative quantities are the mean values.

To confirm the results of microarray, we further validated the selected part of data which we were interested in. The temporal expressions of some chemokines and receptors detected by QuantiGene Plex 2.0 Reagent System were similar with the microarray results (Figure 3). The correlation of quantigene and microarray data was Spearman r = 0.61, P<0.001. CCL2, CCL3, CCL4, CCL7, CXCL9, CXCL10, CCR2 and CCR5 were up-regulated at 3 weeks *p.i,* but subsequently down-regulated at 6 weeks *p.i*. In chronic stages, their expressions were higher than that of 6 weeks *p.i* group. Some other genes such as CCL21, CXCR4, CCR1 and CCR7 exhibited a gradual increase during the infection. These results indicated the approximate coincidence with the data of the microarray experiment.

### CXCL9 and CXCL10 Inhibit the Expressions of Fibrosis Associated Genes in Hepatic Non-parenchymal Cells of Mice Infected with *S.japonicum*

**Figure 4:**
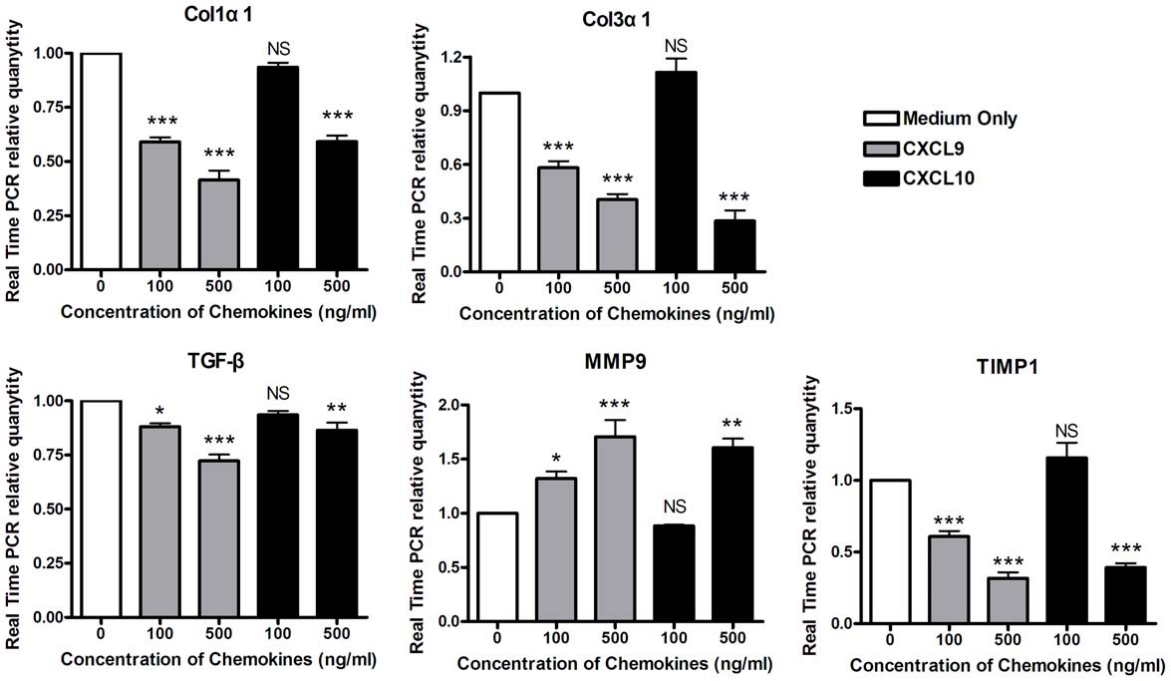
CXCL9 and CXCL10 inhibit the expressions of fibrosis associated genes in liver non-parenchymal cells of mice infected with *S.japonicum*. Liver non-parenchymal cells of mice infected with *S.japonicum* were co-cultured with CXCL9 and CXCL10 respectively for 24 hours. Results of Real Time PCR showed that expressions of Col1α1 (A), Col3α1 (B), TGF-β (C) and TIMP1 (D) were significantly decreased, while MMP9 (E) was increased by stimulations of these two chemokines. Values (Means±SEM) represented the mean of more than three independent experiments. One-Way ANOVA statistical analysis (Newman-Keuls Multiple Comparison Test for analysis of two groups) was used. Each treated group (CXCL9 and CXCL10) was compared with control group (medium only). *, P<0.05; **, P<0.01; ***, P<0.001.

CXCR3 associated chemokines CXCL9 and CXCL10 were reported to be in correlation with liver fibrosis [6], [10], but their roles in liver subpopulations of mice with schistosomiasis were not clear. Hepatic non-parenchymal cells contained heterogeneous cell subpopulations including infiltrative lymphocytes, macrophages, eosinophils, endothelial cell and HSCs, which all played important roles in the pathogenesis of liver diseases. Results of Real Time PCR (Figure 4) showed that after stimulation by different concentrations of CXCL9 or CXCL10 respectively, expressions of Col1α1 (P<0.001), Col3α1 (P<0.001), TIMP1 (P<0.001) and TGF-β (P<0.05) were significantly decreased while MMP9 was increased (P<0.001) in hepatic non-parenchymal cells of mice infected with schistosoma. In addition, we observed that there was not noticeable cell death during the stimulation by CXCL9 and CXCL10. These results showed that both CXCL9 and CXCL10 can inhibit expressions of fibrosis associated genes in non-parenchymal cells of mice infected with *S.japonicum* but up-regulate the expression of MMP9 which may make contribution to fibrotic degradation.

### CXCL9 and CXCL10 Inhibit the Expressions of Fibrosis Associated Genes in Human HSCs Line LX-2 and Primary HSCs of Mice Infected with *S.japonicum*

**Figure 5:**
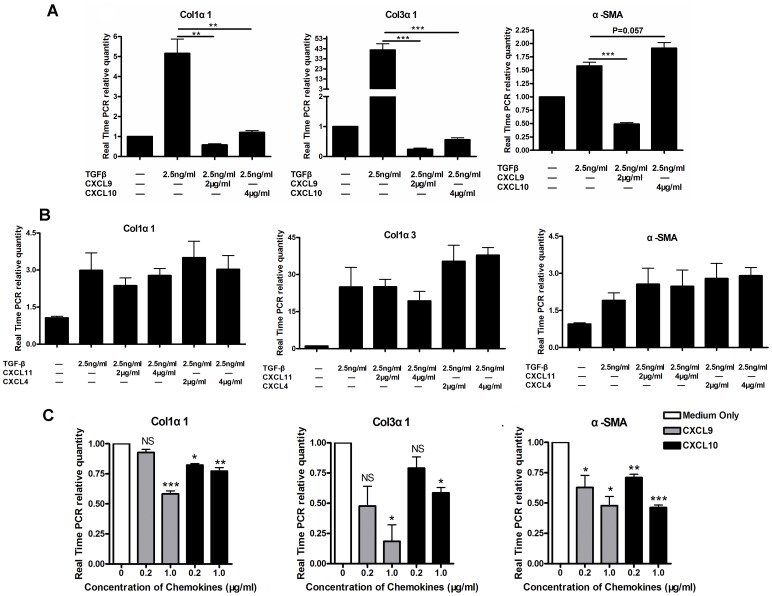
CXCL9 and CXCL10 inhibit the expressions of fibrosis associated genes in human HSCs line LX-2 activated with TGF-β and primary HSCs of mice infected with *S.japonicum*. LX-2 were co-cultured with TGF-β (2.5 ng/ml) for 12 hours, subsequently stimulated with CXCL9 (2 µg/ml), CXCL10 (4 µg/ml), CXCL11 (2 or 4 µg/ml) or CXCL4 (2 or 4 µg/ml) respectively for 24 hours. Primary HSCs were isolated from mice infected with *S.japonicum* for 6 weeks. Freshly isolated HSCs were cultured overnight and followed by stimulations of CXCL9 and CXCL10 respectively for 24 hours. (A) Results of Real Time PCR in human HSCs line LX-2 showed that expressions of Col1α1 and Col3α1 were significantly decreased by stimulations of CXCL9 and CXCL10. Expression of α-SMA was deceased by stimulation of CXCL9, but not CXCL10. P values of two tails T-Test statistical analysis were shown. (B) Results of Real Time PCR in human HSCs line LX-2 showed that expressions of Col1α1, Col3α1 and α-SMA were not significantly decreased by stimulations of CXCL11 and CXCL4 (P>0.05). Each treated group (CXCL11 and CXCL4) was compared with TGF- β treated group. (C) In experiments of primary HSCs, results of Real Time PCR showed that expressions of Col1α1, Col3α1 and α-SMA were all inhibited by CXCL9 and CXCL10. Each treated group (CXCL9 and CXCL10) was compared with control group (medium only). One-Way ANOVA statistical analysis (Newman-Keuls Multiple Comparison Test for analysis of two groups) was used. Values (Means±SEM) represented the mean of more than three independent experiments. *, P<0.05; **, P<0.01; ***, P<0.001. NS, no significance.

To further evaluate the anti-fibrosis effect of CXCL9 and CXCL10, we used human HSCs line LX-2 and the primary HSCs isolated from the mice infected with *S.japonicum*. As shown in Figure 5A, CXCL9 and CXCL10 significantly inhibited the expression of Col1α1 (P<0.01) and Col3α1 (P<0.001) of LX-2, which was activated by TGF-β. Moreover, CXCL9 inhibited the expression of α-SMA (P<0.001), but CXCL10 did not (P = 0.057), suggesting their discordant effects on activation of LX-2. However, other CXCR3 ligands CXCL11 and CXCL4 of 2 or 4 µg/ml concentrations did not show the significant inhibitions on expressions of Col1α1, Col3α1 and α-SMA (P>0.05 compared with TGF-β treated group, Figure 5 B). On the other hand, both CXCL9 and CXCL10 could inhibit the expressions of Col1α1, Col3 α1 and α-SMA on primary HSCs isolated from mice infected with *S.japonicum* significantly (Figure 5 C), showing the same effects on the activation of HSCs. Also, no noticeable cell death was observed during the stimulation by the chemokines which we used in vitro experiment.

### Transcriptional Expressions of Chemokines and Receptors on LX-2 were Down-regulated by Stimulation with SEA

**Figure 6:**
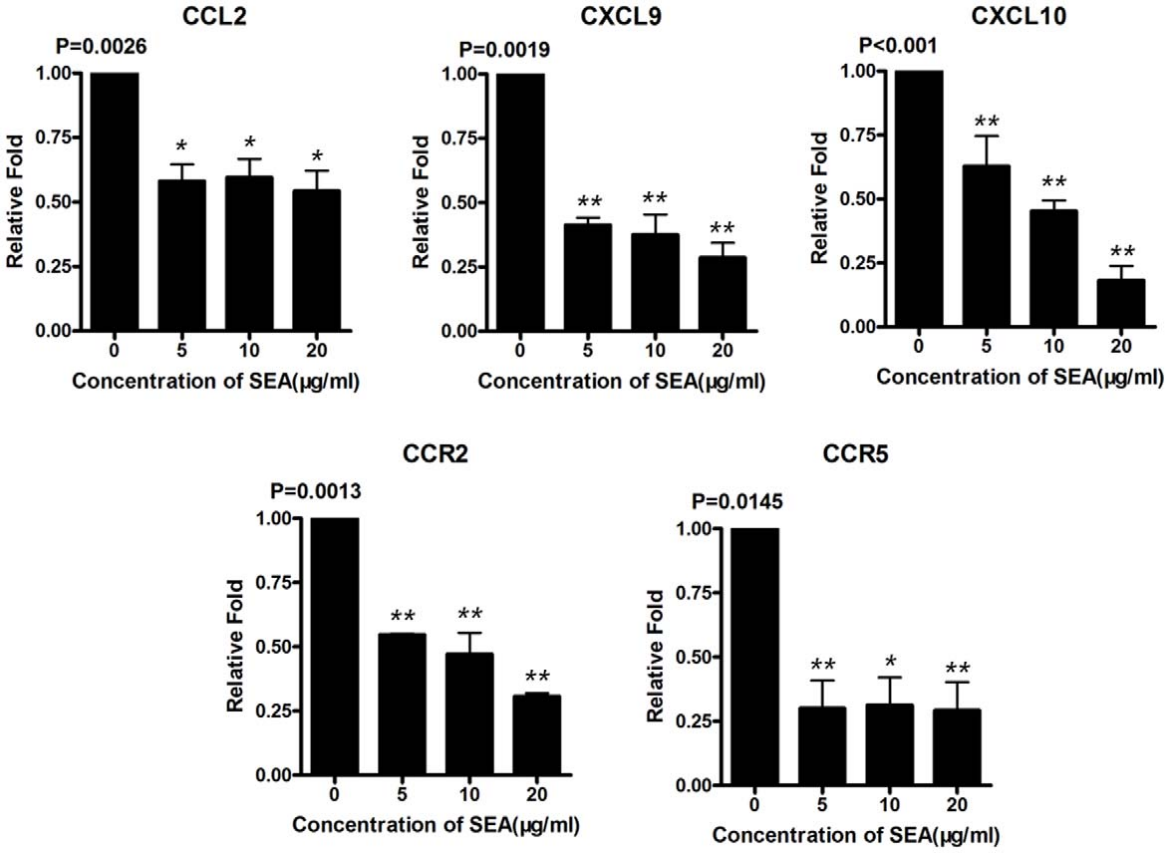
Transcriptional expression of some chemokines and receptors on LX-2 cell line stimulated with SEA. Results of Real Time PCR showed that expressions of CCL2 (P = 0.026), CXCL9 (P = 0.0019), CXCL10 (P<0.001), CCR2 (P = 0.0013) and CCR5 (P = 0.0145) were significantly decreased. Each SEA treated group was compared with control group (medium only). Values (Means±SEM) represented the mean of more than three independent experiments and P values of one-Way ANOVA statistical analysis (Newman-Keuls Multiple Comparison Test for analysis of two groups) were shown. Each treated group (SEA 5, 10, 20 µg/ml) was compared with control group (0 µg/ml). *, P<0.05; **, P<0.01.

It is enigmatic whether the components of schistosoma egg will dysregulate chemokines in HSCs. Therefore, we co-cultured LX-2 with SEA *in vitro* for 24 hours and detected some gene expressions of chemokines. The concentrations of SEA in the culture were according to previous report [13]. Results of Real Time PCR showed that after the stimulation by different concentrations of SEA, the transcriptional expressions of CCL2 (P = 0.0026), CXCL9 (P = 0.0019), CXCL10 (P<0.001), CCR2 (P = 0.0013) and CCR5 (P = 0.0145) on LX-2 were significantly inhibited (Figure 6). These findings demonstrated for the first time that schistosoma egg derived antigens have the capability to directly inhibiting expressions of chemokines on HSCs. The immune inhibition by SEA may contribute to preventing the excessive infiltration of immune cells into the liver and maintaining the immunosuppression during the chronic infection of schistosomiasis.

## Discussion

The crucial role of chemokines and receptors has been highlighted in the liver of mice infected with schistosome [2]. Among the different cellular subpopulations, HSCs are considered to be one of the most important cells in the process of both inflammation and fibrosis. Despite the property of chemotaxis, chemokines and receptors expressed by HSCs mediate the activation and proliferation in liver diseases [21], [22]. Herein, we focused on chemokines expressed on primary HSCs of mice during different stages of schistosomiasis by using microarray. We also detected the dynamic gene expressions after parasitological cure in order to find out the role of chemokines in regeneration of hepatic schistosomiasis. Anti-parasite drug PZQ, which has been described to attenuate the fibrosis [13], was also used as the method of chemotherapeutics.

According to the microarray analysis, several chemokine receptors were located in cluster 1 including CCR2, CCR7, CCR9, CXCR3 and CXCR6, which showed peak expression at 3 weeks *p.i* and the lowest expression at 6 weeks *p.i*. Subsequently, the expressions were slightly increased or not changed during chronic infection. CCR2 and CCR7 are proved to be expressed on HSCs and involved in cell migration, extracellular signal transduction, wound healing and fibrogenic responses in fibrosis models [8]. Our data showed that HSCs exhibited high transcription of CCR2 at 3 weeks *p.i* and suggested that HSCs were primed for activation *via* CCR2 prior to egg deposition. CXCR3 is the only chemokine receptor associated with anti-fibrosis feature on HSCs [10]. However, some other researchers showed that interaction of CXCR3 with its ligands results in the increased activation and chemotaxis of HSCs [23]. These researches suggest that the enigmatic feature of CXCR3 is still not clear and that those conflict observations may be caused by the wide expression of CXCR3 in other cell subpopulations such as Th1 cells and regulatory T cells [24], [25], [26]. Our result showed that CXCR3 expression of HSCs was up-regulated 4.2 fold at 3 weeks *p.i*, but down-regulated at 6 weeks *p.i*. Nevertheless, PZQ treatment significantly induced the expression of CXCR3 in both 12 weeks and 18 weeks *p.i* groups. Therefore, target on CXCR3 might become a prospect therapy in different stages of the schistosomiasis. In addition, CCR9 and CXCR6, which were not reported to be expressed on HSCs in previous research, were expressed in our microarray, and further functional evaluation of them was necessary. Interestingly, expressions of these five receptors in cluster 1 were all increased after PZQ treatment, suggesting that these receptors probably take part in the resolution of the liver fibrosis in the chronic stage after the chemotherapy.

There were 3 chemokine receptors in cluster 2 including CCR1, CXCR4 and CXCR7, which showed sustained up-regulation during both the acute and chronic stages of schistosomiasis mice. CCR1 is actually expressed on primary HSCs in CCl_4_ induced or bile ducts ligation (BDL) mice as a pro-fibrosis receptor [8]. Cluster analysis showed that CCR1 was highly correlated with type I and III collagen, suggesting that it might also promote fibrosis in schistosomiasis [14]. Recently, some research revealed that CXCR4 activation by CXCL12 is profibrogenic through its effects on HSCs activation, fibrogenesis and proliferation [11]. Our data showed that CXCR4 was not changed in acute stage, but up-regulated in chronic stages. These changes suggested that CXCR4 on HSCs might not be anticipated in acute stage of schistosoma infection, but might play a role in activation of HSCs in chronic schistosomiasis. The similarity of temporal expression was also observed in CXCR7, which was the co-receptor with CXCR4 on HSCs [22]. PZQ treatment significantly inhibited the expression of these three receptors, suggesting that they were all involved in activation of HSCs.

The cluster 6 contained two chemokine receptors CCR5 and CX3CR1, which showed persistently low transcriptional levels on HSCs. It is demonstrated that functional CCR5 is expressed on human HSCs [27]. Meanwhile, CCR5 is also expressed on mouse HSCs and plays a pro-fibrosis role [8]. However, conflicting results were also reported in different liver fibrosis models such as schistosomiasis mice. The CCR5^−/−^ mice developed a more severe pathological damage than wild type mice in schistosomiasis mansoni [28]. In our data, expression of CCR5 on HSCs was not changed at 3 weeks *p.i*, but unexpectedly decreased at 6 weeks *p.i*, and maintained a low level during chronic stages. Furthermore, the expressions of CCR5 were increased in PZQ treatment groups. This feature suggested that CCR5 plays a crucial role in fibrotic liver of schistosomiasis as other research reported [28] and the function of CCR5 may be partly attributed to its expression on HSCs. Besides, CCR5 is also expressed on activated T cells and regulatory T cells, which regulate the immune response and control the immunopathology of the schistosomiasis [29]. CX3CR1 and CX3CL1/fractalkine have been implicated in the liver fibrosis [30] and their interaction prevents CCl_4_-induced liver inflammation and fibrosis in the mouse mainly because of the suppression on activation of kupffer cells (KCs) and HSCs [31]. Our data showed a totally reduced expression of CX3CR1 on HSCs during all infection stages and the expression was not significantly changed after PZQ treatment. Since other researches described the up-regulation of CX3CR1 during liver damage [32], our converse result suggested that the transcription of this receptor on HSCs was distinct from that on other CX3CR1^+^ cell subpopulation such as KCs and epithelial cells in damaged liver of schistosomiasis.

Several cytokines such as PDGF, TGF-β as well as chemokines can stimulate activation, migration and proliferation of HSCs by initiate paracrine secretion [3], [5], [22]. HSCs also produce chemokines to regulate immune microenvironment [6]. Our microarray data showed that primary HSCs expressed genes of multiple chemokines during different stages of schistosomiasis mice. After cluster analysis, most of chemokines were in cluster 1 and 2. These chemokines such as CCL2, CCL7, CCL8, CCL11 and CCL12 were demonstrated as pro-fibrosis cytokines in liver diseases [6], [21] and highly expressed during our murine schistosomiasis model, and reduced more or less after PZQ treatment. CCL5 was also detected in our experiment and clustered with monocyte chemotactic protein (MCP) family. However, the increased CCL5 expression after PZQ treatment suggested that it was different from other pro-fibrosis chemokines and might be necessary for the inhibition of inflammation and fibrosis in schistosomiasis as other researches supposed [28]. CXCR3 associated chemokines CXCL9, CXCL10 and CXCL11 have been previously described in HSCs [10], [22], [33]. CXCL9 is the only chemokine defined as anti-fibrosis chemokine for its inhibition of the collagen and TGF-β mRNA and protein levels [10]. Blockage or deficience of CXCL10 leads to the reduced liver fibrosis in CCL_4_ induced liver fibrosis model [34]. However, CXCL10 also shows anti-fibrosis feature in other fibrotic tissues, such as pulmonary [35], [36] and renal tissues [37]. But so far, there are few reports about the function of CXCR3 associated chemokines in different stages of schistosoma infection. Our results of microarray showed that all of CXCR3 associated chemokines were dynamically expressed on HSCs. According to other reports, CXCR3 was also expressed on kupffer cells, T cells, NK cells, endothelial cells, dendritic cells and eosinophils, which were the main compositions of hepatic non-parenchymal cells [38]. *In vitro*, we demonstrated that CXCL9 and CXCL10 inhibited the expression of collagen, TGF-β and TIMP1 of hepatic non-parenchymal cells of mice infected with *S. japonicum*, while increased the gene expression of MMP9, suggesting that they played an anti-fibrosis role in the liver of schistosomiasis. Although other researchers reported the anti-fibrosis role of CXCL9, we further demonstrated that CXCL9 and CXCL10, but not CXCL11 or CXCL4 could decreased the gene expression of the collagen on HSCs line LX-2 activated by TGF-β, which was considered as an important pro-fibrosis mediator in schistosomiasis. The functional distinction of CXCL11 may be due to the different structure [39]. Moreover, the downstream signaling of CXCR3 is not totally unclear and different ligand may activate distinct intracellular signals leading to dissimilar result. Meanwhile, expression of α-SMA was inhibited in LX-2 by CXCL9 but not CXCL10, suggesting the different function of CXCR3 associated chemokines in liver fibrosis and that the various chemokines might affect HSCs *via* non-chemokines receptors as other researchers reported [40]. More importantly, CXCL9 and CXCL10 significantly inhibited the gene expressions of Col1α1, Col3α1 and α-SMA of primary HSCs isolated from 6 weeks post-infected mice, which showed high gene expressions of collagens according to the data of microarray. The inconsistent roles of CXCL10 on α-SMA expressions of LX-2 and primary HSCs revealed that although the use of LX-2, the immortalized cell line, has increased dramatically in recent years on researches relevant to hepatic fibrosis, these cells are indeed different in part from the freshly isolated HSCs [3].

CCL4 and CCL21 were reported in the liver of mice with schistosomiasis [41]. We further demonstrated that they might be produced by HSCs. We detected the expression of CCL4 and CCL21 on HSCs in our experiment and demonstrated that HSCs contributed to production of these two chemokines in the stages of inflammation and fibrosis. Some other chemokines such as CCL3, CCL6, CCL9, CXCL5, CXCL12, CXCL13 and CCL24 are not reported in HSCs. Although most of them are detected in the liver of mice with schistosomiasis [2], [41], whether they are expressed in translational level of HSCs and contribute to the development of schistosomiasis are in need of further investigation.

It is reported that the schistosoma eggs induce downregulation of HSCs activation and fibrogenesis [12]. In our microarray data, the down-regulated expressions of some chemokines on HSCs were obviously seen at 6 weeks *p.i* when the granulomas were severe. Whether SEA affects the transcription of chemokines of HSCs remains unknown. In *in vitro* experiment, we stimulated LX-2 with SEA of various concentrations and demonstrated that SEA inhibited mRNA expression of some chemokines such as CCL2, CXCL9, CXCL10, CCR2 and CCR5 on LX-2. This result suggested that SEA might be the main reason for the down-regulated pattern at the peak of egg granulomas. This interesting alteration of immune microenvironment benefited both host and parasites because sustained excessive immune reaction would cause tissue injury and death of host [42]. Meanwhile, the survival of host was also imperative for the life cycle of parasites.

In conclusion, we present a comprehensive study of chemokines transcriptional profile of HSCs in mouse model of schistosomiasis. HSCs dynamically express multiple chemokines and receptors during schistosomiasis and temporal expressions of distinct chemokines suggest a crucial role in determining the outcome of *S. japonicum* induced pathology. Moreover, pathogenic cure with PZQ significantly attenuates liver fibrosis and changes the expression pattern of some chemokine receptors, suggesting that some receptors are involved in liver fibrosis resolution and may develop into promising therapeutic targets of liver fibrosis in schistosomiasis and other diseases. In addition, we demonstrated that CXCL9 and CXCL10 suppressed the fibrosis associated gene expression in liver non-parenchymal cells and primary HSCs of mice in schistosomiasis and TGF-β -activated human hepatic stellate cells LX-2, suggesting their potential anti-fibrosis role in liver fibrosis. Furthermore, we demonstrate that SEA down-regulates transcriptional expression of some chemokines on HSCs which is an important factor for alteration of immune microenvironment in schistosomiasis liver when the infection is shifted from acute inflammation reaction to chronic stage.

## Supporting Information

Table S1 **Primers of Real Time PCR.**
(PDF)

Table S2 **Evaluation of schistosoma egg granuloma and liver fibrosis in different stages of schistosomiasis.**
(PDF)

Table S3 **Analysis of Top Higher level Biological Functions/Disorders.**
(PDF)

Table S4 **Analysis of Top Canonical Pathways.**
(PDF)
