# Supplementary material for: Gene Profile of Chemokines on Hepatic Stellate Cells of Schistosome-Infected Mice and Antifibrotic Roles of CXCL9/10 on Liver Non-Parenchymal Cells
Source: PLoS One. 2012 Aug 8;7(8):e42490. doi: 10.1371/journal.pone.0042490 (PMC3414521; doi:10.1371/journal.pone.0042490)
Supplement: Table S1 — Primers of Real Time PCR. (PDF) [file pone.0042490.s001.pdf]

human GAPDH	Forward	5’-ATGCTGGCGCTGAGTACGTC-3’
	Reverse	5’-CAGGGGTGCTAAGCAGTTGGT-3’
human CCL2	Forward	5’-CAGCCAGATGCAATCAATGCC-3’
	Reverse	5’-TGGAATCCT GAACCCACTTCT-3’
human CXCL9	Forward	5’-CCAGTAGTGAGAAAGGGTCGC-3’
	Reverse	5’-TGGGGCAAATTGTTTAAGGTCTT-3’
human CXCL10	Forward	5’-GTGGCATTCAAGGAGTACCTC-3’
	Reverse	5’-GCCTTCGATTCTGGATTCAGACA-3’
human CCR2	Forward	5’-GCCTT TTTCACATAGCTCTTGGC-3’
	Reverse	5’-AGGAGTCCTTGTGTAGTCACTTT-3’
human CCR5	Forward	5’-AGGGCTGTGAGGCTTATCTTC-3’
	Reverse	5’-CACCTGCATAGCTTGGTCCA-3’
human α-SMA	Forward	5’- CAGGGCTGTTTTCCCATCCAT-3’
	Reverse	5’- GCCATGTTCTATCGGGTACTTC-3’
human Col1α1	Forward	5’- CGGACGACCTGGTGAGAGA -3’
	Reverse	5’- CATTGTGTCCCCTAATGCCTT-3’
human Col3α1	Forward	5’- TGGTCCCCAAGGTGTCAAAG -3’
	Reverse	5’- GGGGGTCCTGGGTTACCATTA-3’
mouse Col1α1	Forward	5’-ACGTCCTGGTGAAGTTGGTC-3’
	Reverse	5’-CAGGGAAGCCTCTTTCTCCT-3’
mouse Col3α1	Forward	5’-TGGTCCTCAGGGTGTAAAGG-3’
	Reverse	5’-GTCCAGCATCACCTTTTGGT-3’
mouse MMP9	Forward	5’-GCTCATGTACCCGCTGTATAGCT-3’
	Reverse	5’-CAGATACTGGATGCCGTCTATGTC-3’
mouse TIMP1	Forward	5’-TGGGAAATGCCGCAGATATC-3’
	Reverse	5’-TGGGACTTGTGGGCATATCC-3’
mouse GAPDH	Forward	5’-TGGAAAGCTGTGGCGTGAT-3’
	Reverse	5’-TGCTTCACCACCTTCTTGAT-3’
mouse TGF-β	Forward	5’-CACCGGAGAGCCCTGGATA-3’
	Reverse	5’-TGTACAGCTGCCGCACACA-3’
