# Supplementary material for: Gene Profile of Chemokines on Hepatic Stellate Cells of Schistosome-Infected Mice and Antifibrotic Roles of CXCL9/10 on Liver Non-Parenchymal Cells
Source: PLoS One. 2012 Aug 8;7(8):e42490. doi: 10.1371/journal.pone.0042490 (PMC3414521; doi:10.1371/journal.pone.0042490)
Supplement: Table S2 — Evaluation of schistosoma egg granuloma and liver fibrosis in different stages of schistosomiasis. (PDF) [file pone.0042490.s002.pdf]

Supplementary Table2. Evaluation of schistosoma egg granuloma and liver fibrosis in different stages of schistosomiasis

Groups	0 week	3 weeks	6 weeks	12 weeks	18 weeks	12 weeks PZQ	18 weeks PZQ
	Measurement of single schistosoma egg granuloma (μm^2)						
Average of area	0	0	60418.64	48661.99	16899.34	37125.65 *	14073.18
standard deviation	0	0	28091.28	24506.14	11149.82	19512.78	10284.33
	Percentage of Sirius Red areas (%)						
Percentage	0.673	0.684	10.709	18.591	14.294	14.214*	11.571$^{\#}$
standard deviation	0.061	0.058	1.390	1.685	1.442	1.104	1.406

*: $P<0.05$, compared to 12 weeks group

#: $P<0.05$, compared to 18 weeks group
