# Supplementary material for: Gene Profile of Chemokines on Hepatic Stellate Cells of Schistosome-Infected Mice and Antifibrotic Roles of CXCL9/10 on Liver Non-Parenchymal Cells
Source: PLoS One. 2012 Aug 8;7(8):e42490. doi: 10.1371/journal.pone.0042490 (PMC3414521; doi:10.1371/journal.pone.0042490)
Supplement: Table S3 — Analysis of Top Higher level Biological Functions/Disorders. (PDF) [file pone.0042490.s003.pdf]

Cluster	Top Higher level Biological Functions/ Disorders	P-value
1	1. Inflammatory Response	$1.07x10^{-9}$ - $6.72x10^{-3}$
	2. Cancer	$8.76x10^{-8}$ - $6.24x10^{-3}$
	3. Immunological Disease	$9.63x10^{-8}$ - $6.76x10^{-3}$
	4. Inflammatory Disease	$4.40x10^{-7}$ - $6.24x10^{-3}$
	5. Neurological Disease	$4.40x10^{-7}$ - $5.55x10^{-3}$
2	1. Cancer	$8.26x10^{-6}$ - $4.78x10^{-2}$
	2. Inflammatory Response	$1.71x10^{-5}$ - $4.94x10^{-2}$
	3. Immunological Disease	$8.46x10^{-5}$ - $4.31x10^{-2}$
	4. Cardiovascular Disease	$2.38x10^{-4}$ - $4.94x10^{-2}$
	5. Ophthalmic Disease	$3.70x10^{-4}$ - $1.66x10^{-2}$
3	1. Immunological Disease	$7.13x10^{-4}$ - $2.68x10^{-2}$
	2. Ophthalmic Disease	$2.10x10^{-3}$ - $2.10x10^{-3}$
	3. Organismal Injury and Abnormalities	$2.10x10^{-3}$ - $2.68x10^{-2}$
	4. Developmental Disorder	$9.97x10^{-3}$ - $3.95x10^{-2}$
	5. Inflammatory Response	$9.97x10^{-3}$ - $3.91x10^{-2}$
4	1. Inflammatory Response	$7.43x10^{-6}$ - $4.42x10^{-2}$
	2. Connective Tissue Disorders	$3.07x10^{-5}$ - $2.24x10^{-2}$
	3. Inflammatory Disease	$3.07x10^{-5}$ - $4.26x10^{-2}$
	4. Skeletal and Muscular Disorders	$3.07x10^{-5}$ - $3.02x10^{-2}$
	5. Hematological Disease	$4.06x10^{-4}$ - $4.32x10^{-2}$
5	1. Dermatological Diseases and Conditions	$1.04x10^{-4}$ - $4.94x10^{-2}$
	2. Organismal Injury and Abnormalities	$1.04x10^{-4}$ - $4.08x10^{-2}$
	3. Inflammatory Response	$2.43x10^{-4}$ - $4.43x10^{-2}$
	4. Immunological Disease	$8.90x10^{-4}$ - $4.43x10^{-2}$
	5. Genetic Disorder	$1.22x10^{-3}$ - $2.05x10^{-2}$
6	1. Dermatological Diseases and Conditions	$9.47x10^{-4}$ - $3.17x10^{-2}$
	2. Organismal Injury and Abnormalities	$9.47x10^{-4}$ - $4.76x10^{-2}$
	3. Infectious Disease	$1.37x10^{-3}$ - $4.40x10^{-2}$
	4. Inflammatory Response	$1.81x10^{-3}$ - $4.11x10^{-2}$
	5. Cancer	$2.88x10^{-3}$ - $4.70x10^{-2}$
7	1. Organismal Injury and Abnormalities	$7.34x10^{-4}$ – $4.70x10^{-2}$
	2. Psychological Disorders	$1.83x10^{-3}$ - $1.83x10^{-3}$
	3. Cardiovascular Disease	$2.16x10^{-3}$ - $4.70x10^{-2}$
	4. Inflammatory Disease	$2.16x10^{-3}$ - $2.78x10^{-2}$
	5. Inflammatory Response	$2.16x10^{-3}$ - $3.93x10^{-2}$
