# Supplementary material for: Gene Profile of Chemokines on Hepatic Stellate Cells of Schistosome-Infected Mice and Antifibrotic Roles of CXCL9/10 on Liver Non-Parenchymal Cells
Source: PLoS One. 2012 Aug 8;7(8):e42490. doi: 10.1371/journal.pone.0042490 (PMC3414521; doi:10.1371/journal.pone.0042490)
Supplement: Table S4 — Analysis of Top Canonical Pathways. (PDF) [file pone.0042490.s004.pdf]

Cluster	Top Canonical Pathways	P-value
1	1. Cell Cycle Control of Chromosomal Replication	1.14×10^{-7}
	2. Communication between Innate and Adaptive Immune Cells	3.13×10^{-5}
	3. Mismatch Repair in Eukaryotes	5.19×10^{-5}
	4. Altered T Cell and B Cell Signaling in Rheumatoid Arthritis	1.2×10^{-4}
	5. Crosstalk between Dendritic Cells and Natural Killer Cells	2.32×10^{-4}
2	1. Oxidative Phosphorylation	8.72×10^{-20}
	2. Ubiquinone Biosynthesis	2.81×10^{-14}
	3. Mitochondrial Dysfunction	3.02×10^{-13}
	4. HIF1a Signaling	1.32×10^{-7}
	5. Bladder Cancer Signaling	4.56×10^{-5}
3	1. Galactose Metabolism	6.2×10^{-4}
	2. Propanoate Metabolism	1.55×10^{-2}
	3. Butanoate Metabolism	1.66×10^{-2}
	4. PPARa/RXRa Activation	1.72×10^{-2}
	5. Pyruvate Metabolism	2.33×10^{-2}
4	1. Acute Phase Response Signaling	1.02×10^{-3}
	2. PI3K/AKT Signaling	3.03×10^{-3}
	3. Nicotinate and Nicotinamide Metabolism	3.07×10^{-3}
	4. IL-10 Signaling	3.22×10^{-3}
	5. Role of PI3K/AKT Signaling in the Pathogenesis of Influenza	3.82×10^{-3}
5	1. Antigen Presentation Pathway	6.55×10^{-8}
	2. CD28 Signaling in T Helper Cells	1.14×10^{-6}
	3. Type I Diabetes Mellitus Signaling	2.64×10^{-6}
	4. Role of NFAT in Regulation of the Immune Response	9.54×10^{-6}
	5. OX40 Signaling Pathway	1.05×10^{-5}
6	1. Valine, Leucine and Isoleucine Degradation	4.36×10^{-6}
	2. Xenobiotic Metabolism Signaling	2.44×10^{-4}
	3. Lysine Degradation	3.41×10^{-4}
	4. Bile Acid Biosynthesis	5.52×10^{-4}
	5. Molecular Mechanisms of Cancer	7.02×10^{-4}
7	1. Sphingolipid Metabolism	1.22×10^{-4}
	2. N-Glycan Degradation	2.64×10^{-3}
	3. Glutathione Metabolism	2.71×10^{-3}
	4. Starch and Sucrose Metabolism	5.12×10^{-3}
	5. Glutamate Metabolism	6.51×10^{-3}
